# Zebras of all stripes repel biting flies at close range

**DOI:** 10.1038/s41598-022-22333-7

**Published:** 2022-11-03

**Authors:** Kaia J. Tombak, Andrew S. Gersick, Lily V. Reisinger, Brenda Larison, Daniel I. Rubenstein

**Affiliations:** 1 Department of Ecology and Evolutionary Biology, Princeton University, Princeton, NJ USA; 2 Department of Anthropology, Hunter College of the City University of New York, New York, NY USA; 3 Department of Computer Science, San Diego State University, San Diego, CA USA; 4 Leonard Davis School of Gerontology, University of Southern California, Los Angeles, CA USA; 5 Department of Ecology and Evolutionary Biology, University of California, Los Angeles, CA USA; 6 Center for Tropical Research, Institute of the Environment and Sustainability, University of California, Los Angeles, CA USA

**Keywords:** Evolutionary ecology, Evolution

## Abstract

The best-supported hypothesis for why zebras have stripes is that stripes repel biting flies. While this effect is well-established, the *mechanism* behind it remains elusive. Myriad hypotheses have been suggested, but few experiments have helped narrow the field of possible explanations. In addition, the complex visual features of real zebra pelage and the natural range of stripe widths have been largely left out of experimental designs. In paired-choice field experiments in a Kenyan savannah, we found that hungry *Stomoxys* flies released in an enclosure strongly preferred to land on uniform tan impala pelts over striped zebra pelts but exhibited no preference between the pelts of the zebra species with the widest stripes and the narrowest stripes. Our findings confirm that zebra stripes repel biting flies under naturalistic conditions and do so at close range (suggesting that several of the mechanisms hypothesized to operate at a distance are unnecessary for the fly-repulsion effect) but indicate that interspecific variation in stripe width is associated with selection pressures other than biting flies.

## Introduction

The evolutionary origins of zebra stripes have been investigated—and debated—for centuries. The trait is rare, conspicuous, and intensely expressed, and thus appears to beg an adaptationist explanation. However, the utility of a complete coat of densely packed, starkly contrasting black-and-white stripes is not immediately apparent. Unlike many conspicuous visual traits, striped pelage is expressed with comparable intensity in both sexes and is thus unlikely to have arisen through sexual selection alone (although in plains zebras, *Equus quagga*, males have stripes closer to true black than females). Stripes are clearly not aposematic warning signals, nor do they provide camouflage in either the woodland or savannah habitats common across zebra ranges^1,2^. So, striping presents an ideal evolutionary puzzle: a trait so refined it seems it must be “for” something, but one that confers no clear advantage upon its bearers and imposes apparent costs (conspicuousness) that cannot be explained in Zahavian terms.

Scientists have proposed and investigated several possible explanations for the evolution of zebra stripes (reviewed in^3^). The hypotheses suggest various ways in which stripes may provide a social function (species or individual recognition or social cohesion^1,4^), a temperature-regulation benefit^5,6^, an anti-predator effect^7,8^, or an anti-parasite effect^9,10^. There is continued debate over both the merits of individual hypotheses and the likelihood of stripes having arisen via a single driver *vs.* a confluence or alternation of multiple selective pressures^6,11^.

The present study addresses the hypothesis that has thus far received the most empirical support: the anti-parasite hypothesis (also known as the ectoparasite hypothesis^12^). Zebras, like most ungulates, are harassed by tabanid, glossinid and *Stomoxys* species of biting flies, which can inflict significant blood loss, transmit disease, and weaken hosts when fly-avoidance behaviors reduce the host’s feeding rate^9,13,14^. Yet zebras are attacked far less than sympatric ungulates across their African range^15,16^, and also less than other equids^9,17^. Zebras also produce odors that may augment their anti-fly defenses^18^, but so do other sympatric ungulate species^18,19^, and a host of observations and experiments have demonstrated that black-and-white stripes alone are unattractive, or actively repellent to tabanid, glossinid, and *Stomoxys* flies^17,20–23^.

Though the effect of stripes on flies is well-established, the source of the effect remains unexplained. Since Waage’s foundational studies in the 1970s and 1980s^9,24^ most hypotheses have suggested ways that stripes might interfere with the visual and navigational systems of flies, making it harder for them to locate, identify, or successfully land on striped targets. These hypothetical mechanisms can be roughly grouped by the distance (and the attendant phase of a fly’s orientation and landing behavior) at which they would likely operate:

- From *afar*: stripes might make it harder for flies to locate and distinguish zebras from background vegetation, perhaps by breaking up their outline^9^ or varying the way they polarize or reflect light^17,31^ especially from distances at which composite eyes support only low-resolution vision and cannot resolve zebra stripes as clear bands of alternating color on a single host (estimated at > 2.0 m^22^, > 4.4 m^24^, and even > 20 m^25^).
- At *close range* (estimates range from 0.5 to 4.0 m^26^): stripes might interfere with orientation or landing behavior via any of several disruptive or ‘dazzle’-related visual effects^27^. For example, stripes might affect ‘optic flow’, or the fly’s perceived relative motion to its target as it approaches, by creating an illusion of false direction or speed of motion (e.g., via variants of the ‘barber pole’ or ‘wagon wheel’ effects^28^). Alternatively, relative motion to a striped pattern within the visual field may create the perception of self-rotation, inducing the fly’s involuntary ‘optomotor response’ and resulting in an avoidance turn in an effort to stay on a straight course^29^.
- Finally, stripes might cause confusion in the *transition* between long- and short-distance orientation. If zebras appear as blurred gray from a distance and then, at closer range, suddenly resolve into a sequence of floating black and white bars, this abrupt ‘visual transformation’^26^ might disrupt the behavioral sequence that facilitates landing.

Within these categories, hypotheses have proliferated faster than experimental tests of many of the proposed mechanisms. The very active literature on this question has grown in somewhat haphazard fashion, as curious researchers test new possibilities without eliminating old ones^6^. Importantly, few experiments have controlled the distance from which flies are first able to view potential landing sites (but see^23^). While growing evidence supports a mechanism operating at close range^22,26^, failing to restrict the starting distance of the fly means that the full set of possible mechanisms outlined above all remain plausible contributors to most previous results.

Additionally, while many studies have, appropriately, used artificial stimuli to isolate basic effects of color, pattern, brightness, and light polarization of (usually flat) test surfaces, possible contributions of several aspects of natural zebra pelage remain untested. Controlled experiments have used various landing substrates, including striped and solid oil tray traps, sticky plastic, smooth plastic^17^, cloth (Experiment 2 in^22^), horse blankets or sheets^26^, and paint on live animals^30^. These have all clearly demonstrated a broadly replicable visual effect: stripes, and some *other* juxtapositions of black and white (e.g., checkerboard patterns^26^), repel flies. However, insofar as specific features of zebra pelage factor into proposed mechanisms of fly repellence—the reflective properties of “smooth, shiny” coats^31^; the orientation of the stripes^17,32^; the light-polarizing effects of black and white hair vs. background vegetation^25^; and the complex structure of hair^25^—there is a need for more experiments that present natural targets to wild flies (but see^22,33^). Similarly, most experiments have compared landing preferences between black-and-white striped, solid black, solid white, and occasionally solid grey substrates, which have served as important controls for determining that light polarization, rather than a combination of polarization and brightness, is sufficient to induce the effect of stripe avoidance^17^. However, it is now time to refocus on the original question by presenting flies with more realistic choices. Since biting flies seeking a bloodmeal on the African savannah seldom encounter solid black hosts, and even more rarely solid white hosts, landing choices should be compared between zebra stripes and common coat colors of sympatric mammals, namely various shades of brown. Further, tabanid, glossinid, and *Stomoxys* flies all avoid landing on stripes that are the same width or narrower than the widest zebra stripes ^17,23^, and there is some evidence that narrower stripes are even more repellent to tabanids^17^. This pattern is potentially significant in the application of the anti-parasite hypothesis to an adaptive explanation for the striking variation in stripe width across zebra species and between the different areas of the body on individual zebras^22^, but must first be confirmed with experiments using real zebra pelage.

Here, we present a simple experiment designed to address each of these gaps in the literature on the anti-fly benefits of zebra stripes. In this field experiment, the landing choices of flies were tested entirely within the range at which all estimates agree flies should be able to perceive the presented stripes (< 1.0 m). This restriction enabled us to determine whether hypothesized mechanisms that act at close range are sufficient to produce the fly-repulsion effect, potentially revealing other mechanisms to be unnecessary (all mechanisms that operate at or beyond the distance at which zebra stripes can be resolved). Our paired-choice experiments used real animal pelts, both striped (zebra spp.) and solid tan (impala; *Aepyceros melampus*), mimicking the actual choices made by flies cruising the African savannah. Using pelts over live animals allowed us to remove potentially interacting effects of fly-deterrent *behavior* (tail-switching, stomping, skin rippling, and other movement) that are less relevant to specific questions about the evolution of zebra stripes. We further isolated *visual* properties of the pelts by salt-curing the hides, a well-established gentle preservation method that (a) minimally impacts the specific luminance, reflectance, and pigment saturation, as well as the depth, density, and structure of animal hair, while (b) minimizing the persistence of volatile olfactory compounds that might be independently aversive to flies^34^. To further minimize the potential confound of odor, we chose impala skin as the alternative landing substrate to zebra pelts; like zebras, impala are consistently unattractive hosts for biting flies^19,35,36^. Finally, we tested pelts from the zebra species with the widest stripes overall (the plains zebra, the only species with wide stripes across most of its body^17^) and the narrowest stripes (the Grevy’s zebra, *Equus grevyi*) under the same conditions, to investigate whether cross-species variation in stripe width could represent differences in their abilities to repel flies.

## Results

**Figure 1:**
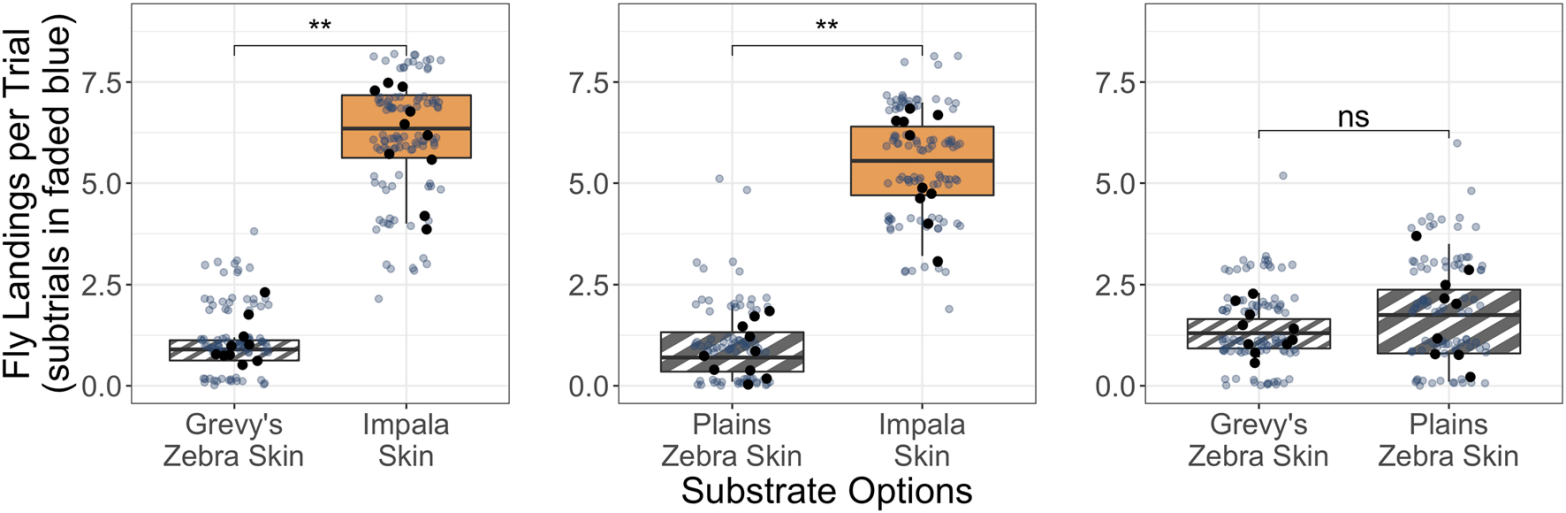
Number of fly landings per trial on zebra and impala skins for each paired-choice treatment (statistics run on mean landings per trial, shown by large black points, which were mean counts across subtrials within a trial session, shown in faded navy points). *ns* no significant difference, **p < 0.01.

The flies had a strong preference for impala skin over zebra skins of either species (Grevy’s zebra ‘GZ’ vs. impala ‘IM’: V = 0, *p* < 0.01, N = 10 trials; plains zebra ‘PZ’ vs. IM: V = 0, *p* < 0.01, N = 10 trials). However, there was no difference in preference between the two zebra species (V = 19.5, *p* = 0.44, N = 10 trials; Fig. 1). We found no left/right bias in our arena throughout the trials (V = 238, *p* = 0.64).

When flies landed on zebra skin of either species, they were much more likely to land on a black stripe than on a white stripe (a fly would land on a black stripe 75.4% of the time when landing on a zebra skin, N = 483 landings on zebra skin for which the stripe color was recorded). This effect was remarkably similar between the two zebra species (187 landings on black PZ stripes and 177 on black GZ stripes vs. 59 landings on white PZ stripes and 60 on white GZ stripes).

**Figure 2:**
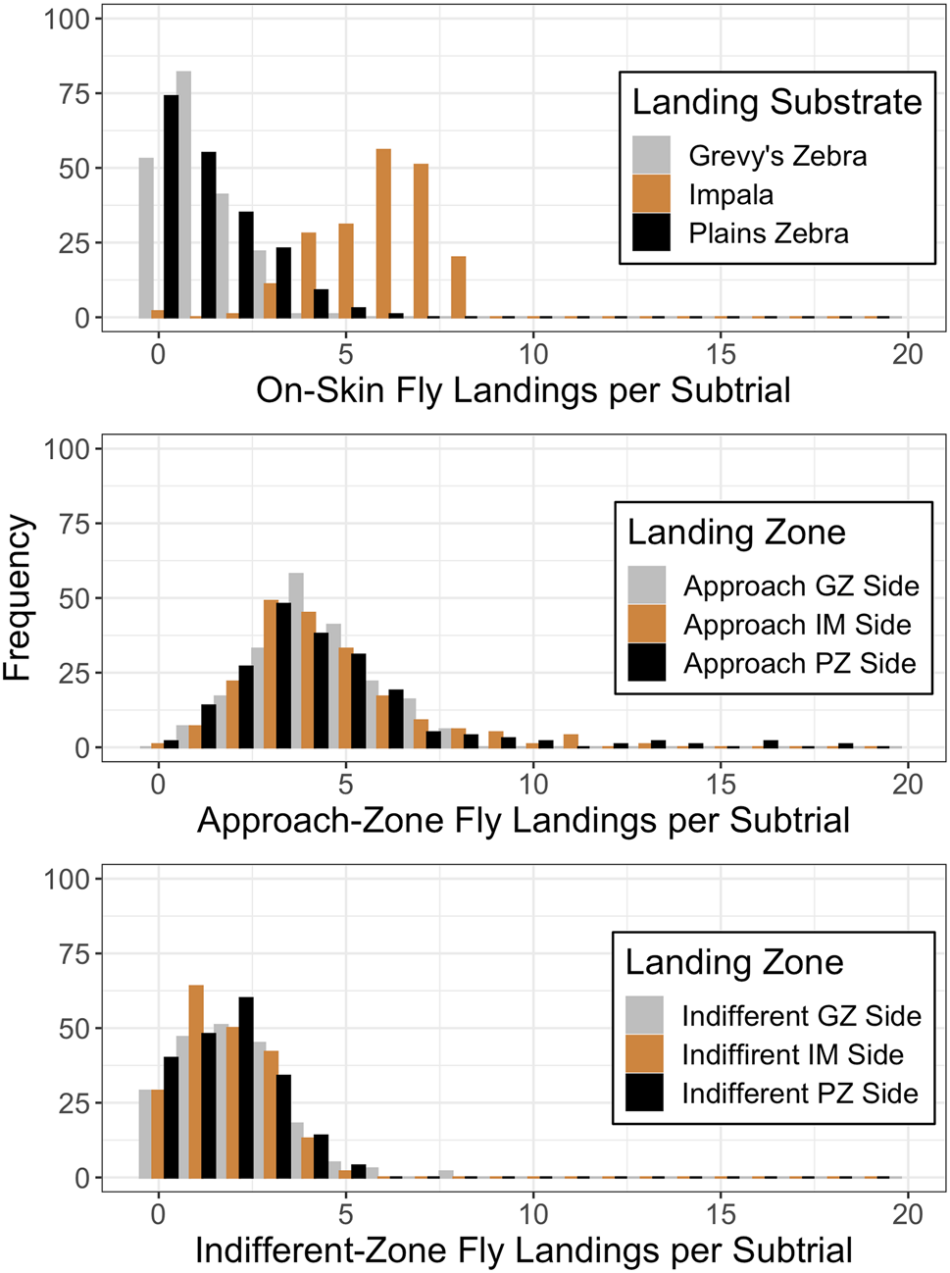
Frequency distributions of landings per subtrial on the different skins and in the off-skin zones within the arena in the paired choice experiments.

Across subtrials (each subtrial representing a landing choice after a disturbance), most flies landed in the approach zones on either side of the arena (N = 2813; 869 of them on the GZ side, 868 of them on the IM side, and 849 of them on the PZ side), followed by the ‘indifferent’ zone near the center (N = 1303; 415 of them on the GZ side of the petri dish, 352 of them on the IM side, and 346 of them on the PZ side). The fly count on impala skin was highest after that (N = 1155) while landings on zebra skins occurred at roughly one quarter of this frequency (N = 239 on GZ skin and 251 on PZ skin; note that these differ from the sum of landings on black and white stripes for each zebra species because the color of the stripe for each landing could not always be recorded in time for every fly). In each subtrial, very few flies tended to choose to land on zebra skins, but relatively few flies landed near the center (‘indifferent’ zone) as well, suggesting that there was motivation to move towards one of the ends of the arena (Fig. 2). Flies that landed in any intermediate areas of the arena were as likely to land on the side closer to a zebra skin as on the side closer to the impala skin, including landings in the approach zone within 30 cm of a skin (Fig. 2, second panel).

## Discussion

For a single trait of a relatively small clade, zebra stripes have sparked the curiosity of a significant number of researchers and catalyzed a large and active body of research. Within this literature, there are legitimate disagreements over competing hypotheses concerning both the evolutionary drivers of striping, and the proximate mechanisms by which stripes benefit zebras. The results of the present paper help prune the set of plausible hypotheses on the mechanism by which zebra stripes repel flies.

Our experiments first addressed questions about the range at which a visual mechanism might discourage flies from landing on zebras. Flies in our experiment first encountered targets at close range (< 1.0 m), and all interactions occurred within that range. The strong preference flies showed for impala pelt over zebra pelts suggests that a mechanism operating within the distance at which flies perceive stripes is sufficient to deter them from landing. Visual effects occurring at long distances (i.e., where flies have trouble distinguishing zebras from background landscapes)—or sudden ‘visual transformations’ as flies approach and stripes come into view—may still occur. They are simply not necessary to repel flies from landing. Some prior studies have observed apparent close-range effects, but did not constrain the starting distance of approaching flies^22,26^. Our results demonstrate that a close-range mechanism can operate without any long-range or transitional component. Further, we found that flies were no *less* likely to approach within 30 cm of zebra skins than impala skins, constraining the likely action of a repellent effect to within distances of 30 cm or less. This accords with How et al.^26^, who found that the median closest distance to which flies approached striped and solid horse blankets was ~ 18 cm and 6 cm, respectively. While we found that stripes are most likely to repel flies at very close range, we did not commonly observe them to ‘crash land’ or bump off of zebra skins as described anecdotally by Caro et al.^22^.

Our results also begin to address an imbalance in the literature, between hypotheses that invoke fine-grained details of natural zebra-fly interactions and experiments that test simplified, artificial visual targets. While artificial targets might amplify the visual effects of coloration by isolating and intensifying the visual signal, our results demonstrate a strong effect using targets that retain the structural complexity of natural pelage, while still controlling for other features of live-animal interactions in the wild—e.g., odor and anti-fly behaviors—that can also confound tests of a visual phenomenon. While it is possible for some residual differences in odor to persist after salt-curing the hides, our finding that flies landed equally frequently in the approach zones close to zebra and impala skins indicates that a visual effect acting at very close range is more likely than odor to regulate landing preferences on the skins. The jagged boundaries between black and white stripes on natural pelage, their curved shape, the intricate patterns on some areas of natural pelts, and the light-scattering effects of hair, all distinguish real zebra pelts from the artificial stimuli tested in most experiments to date. Any or all of these natural features might have altered the visual effects of stripes that have been previously demonstrated in experiments with clear, straight-banded artificial stimuli. Instead, our results match those of past experimental work, thereby lending biological validity to the full body of experimental evidence for the fly-repellent effects of zebra stripes.

Many experiments using artificial targets have tested fly landing behavior on striped surfaces compared to solid black or solid white surfaces. The resulting effect sizes have varied widely, and some have even shown a lower preference for solid white surfaces than for striped ones (e.g., Experiment 2 in^17^). Our comparison of striped and brown targets is more relevant to the actual choices flies must make in the wild. Solid white animals are very rare on the African savannah, but tan, brown, and dun-colored coats are common. We confirm that, given a choice between natural brown pelts and zebra pelts, biting flies show a strong preference to land on the former. When they do land on striped substrates, flies have been found to prefer to land on black stripes over white stripes^17^, and we confirm that this is also true for natural zebra skins and for the full range of stripe widths seen across zebra species.

Using real pelts was perhaps most important in our comparison of landing preferences between Grevy’s and plains zebra skins. Counter to previous findings from experiments using oil tray traps^17^, we found no difference in the frequency of fly landings on skins with wider stripes or narrower stripes, suggesting that interspecific variation seen in zebra stripe width does not result in a difference in how well different zebra species repel biting flies. The discrepancy between our results and those from prior work may come from the fact that the stripe boundaries are not as clear-cut and stark on pelts as on painted surfaces and the hair itself is likely to scatter light, perhaps lessening the distinction between stripes of 1–2 cm and 2–4 cm in width. This finding also weighs against the hypothesized ‘optomotor response’ mechanism, which would predict that wider stripes passing through a moving fly’s visual field would create a slower perceived self-rotation than narrower stripes and should therefore induce a weaker repellent effect. However, it may be that the avoidance response would simply be slower in the face of wider stripes, and the rate of actual landing on the two zebra species may still be comparable.

Why the Grevy’s zebra evolved the narrowest stripes of all zebras, and the plains zebra the widest stripes, remains an open question. The third extant zebra species—the mountain zebra, *Equus zebra*—has stripes mostly of intermediate width and is the evolutionary outgroup among zebras^37^. Solid brown coloration even replaced stripes across the flanks and rump of the southern-most subspecies of the plains zebra, the recently-extinct quagga (*Equus quagga quagga*), illustrating the wide variation in zebra striping across environments. If, as our results indicate, stripes that span the range of interspecific stripe width variation repel flies to the same extent, adaptive explanations for stripe width variation would have to pertain to some other selective pressure. The native range of the Grevy’s zebra in the Horn of Africa is more arid than that of the plains zebra^38^, and the Grevy’s zebra forms loose bonds in unstable groups while plains zebras form stable family groups^39,40^. This may implicate some of the other hypotheses for the evolution of zebra stripes (e.g., those positing a thermoregulatory, anti-predator, or social function of stripes) to explain how this trait may have been refined within the zebra clade, as opposed to how it initially arose. It is also possible that the costs of starkly contrasting stripes (e.g., their conspicuousness) weigh differently on the different zebra species in their native habitats and that stripe width variation reflects the mitigation of these costs to different degrees.

Our finding that stripe width did not alter fly repulsion also addresses proposed explanations for the variation in stripe width between different parts of the zebra body. In all zebra species, stripes are the narrowest on the face and legs^17,41^. This has been suggested to align with the anti-parasite function of zebra stripes, as flies will often take flight from vegetation and cruise close to the ground, encountering the legs or face of a grazing zebra before other parts of the body^41–43^. But if narrower stripes are not better at deflecting biting flies, why are stripes narrowest in these regions of the body? There may be an adaptive reason for this, or it may simply be a matter of ontogeny, such as an artifact of differences in the spacing of differentiated melanocytes between more proximal parts of the body and the extremities^44–46^.

Our experiments were intended to facilitate future investigations into the origins of zebra striping in two ways: by rendering some categories of mechanistic hypotheses unnecessary and by reintroducing the natural structural complexity of real animal pelts into a body of evidence that has largely focused on visually simplified, artificial stimuli and conditions. We believe our results strongly implicate a visual mechanism that operates at distances within the range at which dipteran eyes can resolve stripes, and one in which all stripes within the natural range of stripe width expressed by zebras have an equally strong effect on fly landing preferences. However, we remain in the dark as to precisely what visual, perceptual or cognitive mechanism drives the avoidance of striped pelage as flies make landing choices. Clearly, there is still a need for creative investigations of stripes from a fly’s-eye view to uncover what makes them visually aversive or challenging for flies to approach. Furthermore, if variation in the width of stripes on natural pelage does not compromise their anti-fly properties, it seems that researchers will have to look to other sources of selection pressure for adaptive explanations for cross-species variation in zebra stripes.

## Methods

Sections of zebra and impala skin were extracted from carcasses found at Mpala Research Centre (MRC), Laikipia District, Kenya. We took a ~ 400 cm^2^ piece from the side of the rump from one plains zebra and one Grevy’s zebra (SI1), and one section from the flank of an impala (since the rump of the impala was not large enough to match the skin sections from the zebras), all found dead in the field. Each of these squares was scraped free of any attached muscle and fat, cured with salt, and sun-dried at the research center.

We set Nzi traps^47^ to collect local biting flies next to a camel corral close to the research center, baited with a bucket of a mixture of fresh camel urine and milk at the center of the trap. Traps were set for 6–24 h until a sufficient number of flies was collected for an experiment, and flies were harvested from traps 12 h before the morning experiments. To ensure that all flies were hungry, they were kept in fly boxes outside overnight (almost all were alive the next morning). Researchers from the International Centre for Insect Physiology and Ecology (ICIPE) were on site to verify that the flies were *Stomoxys* species.

**Figure 3:**
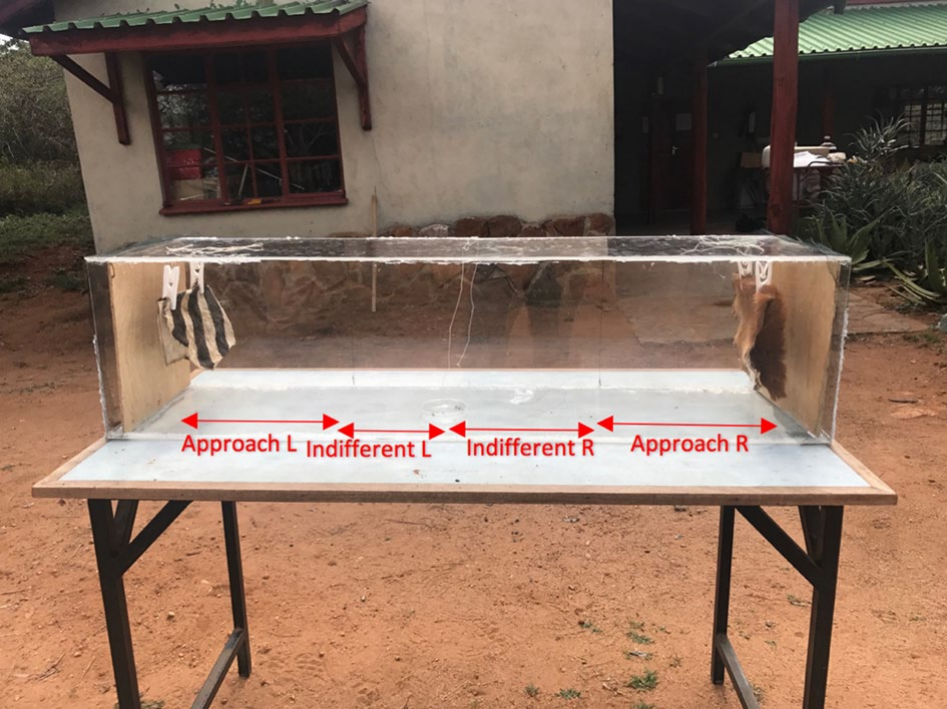
Experimental set-up for a plains zebra vs. impala skin trial. Landings were recorded as having occurred on a skin surface, within the approach zone of 30 cm from the skin, or in the indifferent zone of 30–60 cm from the skin on either side of the petri dish. Photo credit: LVR.

The experimental arena comprised a four-sided rectangular Plexiglas box (120 cm × 30 cm × 30 cm) with a removable square piece of wood wedged in at each end to serve as the background for an animal skin (the ends prevented flies from escaping but were not so tightly sealed as to limit the oxygen in the arena; Fig. 3). Ahead of each trial, an animal skin was hung just in front of each wooden square end with clothespins attached with fishing line to the top of the box.

Fly landing experiments were conducted at MRC over a short time period to maximize the uniformity of conditions, 20–22 July, 2017. Trials were run in the mornings (8:30–10:00) and in the afternoons (15:00–16:30), as these are the times of day when *Stomoxys* flies are active^48^. A covered petri dish containing 15–25 flies was placed in the center of the arena. Trials comprised a series of “landing-choice subtrials”. At the beginning of the trial, the cover was lifted with a fishing line threaded through a small hole at the top of the box to release the flies, and a stopwatch was started. When all flies had settled (typically within 5 s), the position of each was noted as being within one of six zones in the arena (having landed on either skin, having approached a skin within the ‘approach’ zone of 30 cm, or having remained in the ‘indifferent’ zone between 30 and 60 cm from a skin on either side of the arena’s midpoint; Fig. 3). For the flies that landed on a zebra skin, we noted whether they landed on a black or white stripe whenever possible. After a one-minute interval—allowing time for all flies to land and for human observers to note the locations of all fly landings—the top of the box was tapped several times such that all flies took flight, initiating the next landing-choice subtrial, after which all landing positions were recorded again. This cycle was repeated for a total of 10 subtrials within each trial. The landing choices of any resting flies that took off and alighted again within a subtrial were also recorded. The researcher approached the experimental arena quietly and slowly to avoid disturbing the flies and stood next to the midpoint of the arena, aligned with the petri dish, at all times during observations.

The paired choice combinations tested comprised a Grevy’s zebra skin *vs.* a plains zebra skin, a plains zebra skin *vs.* an impala skin, and a Grevy’s zebra skin *vs.* an impala skin (N = 10 trials and 100 subtrials for each comparison). Landing counts on each skin in each subtrial were combined into averages per trial to avoid pseudoreplication due to the non-independence of a fly’s position between subtrials. These averages were compared between skins for every choice combination using paired Wilcoxon Rank Sum tests (paired by trial). Note that in a Wilcoxon Rank Sum test, a W = 0 (or V = 0 in the case of a paired test) result indicates that all observations from one group rank above all observations of the other group (a perfect difference in ranks). The skins were alternated between trials such that each skin was tested at both ends of the arena for each choice combination to enable the statistical control for a bias for which side of the arena was chosen by the flies (using a paired Wilcoxon Rank Sum test between the mean number of times per trial that the left *vs.* right side of the arena was chosen by a landing fly) (Supplementary Information).

## Supplementary Information

Supplementary Information.

## Data Availability

All data associated with this manuscript are available on Dryad (10.5061/dryad.gb5mkkwtd) and the script is available on Zenodo (10.5281/zenodo.7402731).
