# Author Correction: Zebras of all stripes repel biting flies at close range

**DOI:** 10.1038/s41598-023-28782-y

**Published:** 2023-01-31

**Authors:** Kaia J. Tombak, Andrew S. Gersick, Lily V. Reisinger, Brenda Larison, Daniel I. Rubenstein

**Affiliations:** 1 Department of Ecology and Evolutionary Biology, Princeton University, Princeton, NJ USA; 2 Department of Anthropology, Hunter College of the City University of New York, New York, NY USA; 3 Department of Computer Science, San Diego State University, San Diego, CA USA; 4 Leonard Davis School of Gerontology, University of Southern California, Los Angeles, CA USA; 5 Department of Ecology and Evolutionary Biology, University of California, Los Angeles, CA USA; 6 Center for Tropical Research, Institute of the Environment and Sustainability, University of California, Los Angeles, CA USA

Correction to: *Scientific Reports* 10.1038/s41598-022-22333-7, published online 03 November 2022

The original version of this Article contained errors in the Methods and Data availability sections.

“We took a ~ 30 cm^2^ piece from the side of the rump from one plains zebra and one Grevy’s zebra (SI1), and one section from the flank of an impala (since the rump of the impala was not large enough to match the skin sections from the zebras), all found dead in the field. Each of these squares was scraped free of any attached muscle and fat, cured with salt, and sun-dried at the research center.”

now reads:

“We took a ~ 400 cm^2^ piece from the side of the rump from one plains zebra and one Grevy’s zebra (SI1), and one section from the flank of an impala (since the rump of the impala was not large enough to match the skin sections from the zebras), all found dead in the field. Each of these squares was scraped free of any attached muscle and fat, cured with salt, and sun-dried at the research center.”

In addition, in the Data availability section,

“All data associated with this manuscript will be available on Dryad upon acceptance.”

now reads:

“All data associated with this manuscript are available on Dryad (https://doi.org/10.5061/dryad.gb5mkkwtd) and the script is available on Zenodo (https://doi.org/10.5281/zenodo.7402731).”

The original Article has been corrected.

