# Supplementary material for: Zebras of all stripes repel biting flies at close range
Source: Sci Rep. 2022 Nov 3;12:18617. doi: 10.1038/s41598-022-22333-7 (PMC9633588; doi:10.1038/s41598-022-22333-7)

Zebras of all stripes repel biting flies at close range

Kaia J. Tombak*#[1,2], Andrew S. Gersick#[1,3], Lily V. Reisinger[1,4], Brenda Larison[5,6], Daniel I. Rubenstein[1]

#These authors contributed equally to this study.

SI1: The plains zebra (left) and Grevy's zebra (right) pelts used in our experiments. Photo credit: Rosemary Warungu.

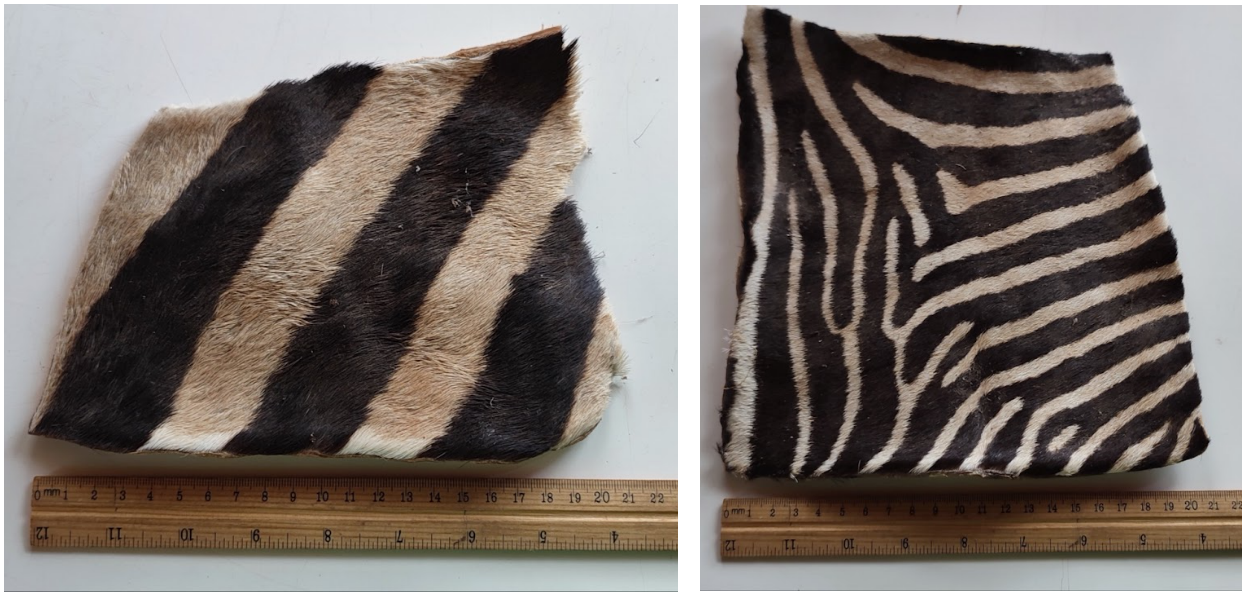

Supplement: Supplementary file 1 — Supplementary Information. [file 41598_2022_22333_MOESM1_ESM.pdf]
